# The Conceptualization and Measurement of Comorbidity: A Review of the Interprofessional Discourse

**DOI:** 10.1155/2013/192782

**Published:** 2013-09-25

**Authors:** Salimah H. Meghani, Harleah G. Buck, Victoria Vaughan Dickson, Marilyn J. Hammer, Eneida Rejane Rabelo-Silva, Robyn Clark, Mary D. Naylor

**Affiliations:** ^1^Department of Biobehavioral Health Sciences, NewCourtland Center for Transitions & Health, University of Pennsylvania, Room 337 Fagin Hall, 418 Curie Boulevard, Philadelphia, PA 19104-4217, USA; ^2^NewCourtland Center for Transitions & Health, University of Pennsylvania, The Pennsylvania State University, University Park, PA 16802, USA; ^3^New York University, New York, NY 10003, USA; ^4^Federal University of Rio Grande do Sul, School of Nursing, 90040-060 Porto Alegre, RS, Brazil; ^5^School of Nursing & Midwifery, Flinders University, Adelaide, South Australia, SA 5001, Australia; ^6^NewCourtland Center for Transitions and Health, University of Pennsylvania, Philadelphia, PA 19109-4217, USA

## Abstract

*Background*. Chronic medical conditions often occur in combination. Understanding underlying mechanisms causing diseases and their interactions may make it possible to address multiple complex conditions with single or consolidated treatment approaches and improve patients' health outcomes while reducing costs. *Objectives*. We present a synthesis of the current interprofessional discourse on the issues surrounding comorbidities. *Methods*. A targeted review of the literature was conducted using published editorials, commentaries, and review articles. *Results*. Errors in conceptualization and measurement plague our current understanding of comorbidities. Two potential paths to generating knowledge involve the use of etiological or epidemiological approach. An etiological approach investigates the risk factors and underlying mechanisms potentially leading to consolidation of diagnosis and treatments. Because of the rudimentary stage of knowledge development in this area, this approach will require time and significant research investments. In contrast, the epidemiological approach relies on statistical identification of disease entities that cooccur beyond random chance; this approach carries an accompanying risk of diagnostic and treatment proliferation. *Discussion*. The concept of comorbidity, its nature, and measurement is in need of meaningful debate by the scientific and clinical communities. Recommendations in the domains of conceptualization, research, and measurement are discussed.

## 1. Introduction

Chronic medical conditions rarely occur in isolation but rather in combination, as comorbidities. Successful management of chronic conditions is associated with complex treatment regimens and requires adequate self-care by the patient of all comorbid conditions. Improving self-care among patients with chronic illness therefore necessitates a better understanding of the complexities of comorbidity. 

While the concept of comorbidity was introduced in medicine almost four decades ago [1], its scientific underpinnings remain underdeveloped with consequent uncritical use and application of this concept in research and practice [2, 3]. The difficult issue of comorbidities has been appropriately referred to in the literature as a “puzzle,” “tapestry” [4], “Gordian Knot” [5], and something that embodies “dizzying” levels of complexity [6]. Thus the concept of comorbidity remains in continued need of discourse and development. Recently, both research and clinical communities have begun to pay close attention to the complexity of comorbidities in an attempt to appreciate its scope and utility for enhancing practice and patient outcomes [7–9]. Specific calls have been made for changes for the multimorbid in (1) how quality is measured, (2) how health care is delivered and paid for, and (3) informed clinical decision making [10, 11]. But comorbidity continues to be plagued by two critical errors rooted in the conceptualization and measurement of the concept. Because of these errors, any current definition of comorbidity should be endorsed carefully. Furthermore, the science cannot advance until they are addressed. 

To better understand and delineate these conceptualization and measurement issues, an integrative review of the current interprofessional discourse surrounding comorbidity was conducted using published editorials, commentaries, and review articles. A literature search was carried out using ISI Web of Science (Science Citation Index Expanded and Social Sciences Citation Index). Review and editorial materials published in the English language between 1999 and 2012 were searched using the terms “comorbid,” “comorbidity,” and “comorbidities” in the title, resulting in 1,354 titles. Meeting abstracts (*n* = 526) and proceeding papers (*n* = 47) were excluded, yielding 781 articles. Pediatric (*n* = 44) and empiric, disease-specific articles were excluded, yielding a final set of 29 articles. This resulted in a synthesis of the current conceptual and measurement issues surrounding comorbidities and an offering of recommendations with potential to address these issues. Future directions were identified in the conceptual, measurement, and analytical domains. 

## 2. Errors of Conceptualization 

Correct conceptualization of comorbidity is a necessary precondition for knowledge development. The correct ascertainment of comorbidity inevitably depends upon the correct identification of the underlying etiology. The etiological science is constantly evolving and will always remain imperfect. In the absence of sound etiological grounding, the science of comorbidity has been criticized for being atheoretical or without a theoretical base [12, 13]. Four problems currently confound the advancement of comorbidity conceptualization—the presence of heterogeneous definitions, an inadequate nosological system, a lack of modeling of the dynamic patterns of relationships between chronic conditions, and an atheoretical understanding of the causes and predictors of comorbidity.

### 2.1. Problem Number 1—Heterogeneous Definitions

**Figure 1:**
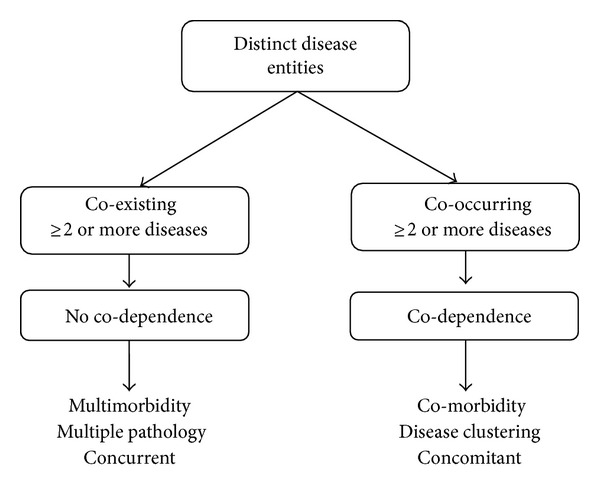
Multimorbidity versus comorbidity (illustration of conceptual problem no. 1).

The term comorbidity was introduced by Feinstein in 1970 to signify a “distinct additional clinical entity” occurring in the setting of an index disease [1]. The term has been used loosely in the literature to imply either “coexisting” diseases or “cooccurring” diseases (Figure 1). Although often used interchangeably, important distinctions exist; for instance, the simultaneous presence of multiple health conditions is also termed “coexisting diseases,” “multiple pathology,” and “multimorbidity” when no single condition can be identified as an index disease [4, 14]. On the other hand, comorbidities are termed “cooccurring diseases,” “concomitant diseases,” and “disease clustering” when diseases cooccur at a significantly higher rate than expected by chance alone [14]. Thus, the existing terms used to denote comorbidities have distinct conceptualizations and scientific implications while still being used interchangeably. 

### 2.2. Problem Number 2—Inadequate Nosological System

The bulk of debate on the nature of comorbidities lies in the domain of nosology or disease classification. This discourse centers on the teasing part of real or true comorbidities from artifacts or spurious comorbidities. To qualify as a comorbid condition, Feinstein argued that each disease must represent a “distinct” disease/clinical entity with unique pathophysiology, course, and response to treatment while sharing a common diathesis/etiology [15]. This is where the conceptualization of comorbidity gets murky, as limited nosological systems challenge the very foundation of the comorbidity designation. 

**Figure 2:**
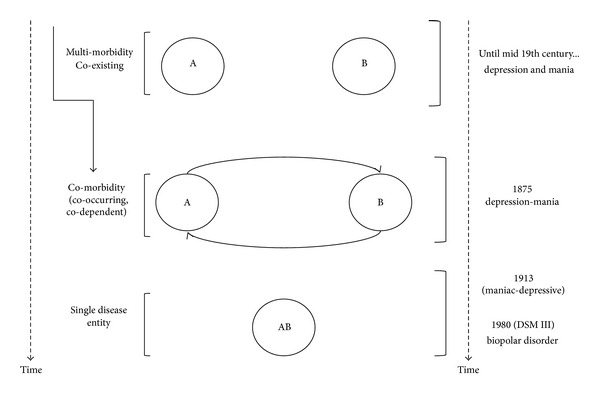
The relation between nosology, chronology and evolving science (illustration of conceptual problem no. 2).

The designation of a valid clinical entity (or taxon disease) assumes that the diagnostic nosology is a concrete science, which is far from the case. There have been several iterations of both the Diagnostic and Statistical Manual of Mental Disorders (DSM) and International Statistical Classification of Diseases (ICD) with earlier versions differing significantly from current ones. This is epitomized in the recognition of depression and mania as “two distinct” clinical entities until late 19th century to a “single” disease, bipolar disorder, in the DSM-III (see Figure 2). Further, the “operational rules” used in the construction of the DSM creates a clinical scenario of diagnostic proliferation when diseases may actually be an extension of the same underlying process [10, 12, 13]. For instance, anxiety is frequently present in individuals with depression; however, the rule in the DSM does not allow occurrence of the same symptom in more than one disorder, resulting in the creation of additional DSM diagnostic categories such as “mixed depressive-anxiety” [12, 13]. Thus, concomitance of two or more diagnoses may indicate either the presence of distinct clinical entities or point to multiple manifestations of a single clinical entity [12, 13, 16, 17]. Contrary to the DSM, the ICD classification allows for similar symptoms or indicator patterns to appear in more than one disorder. Diseases with different etiologies that produce similar pathology and symptoms are defined as two separate diseases in the ICD classification. However, the knowledge of diseases with shared etiologies, but disparate clinical symptoms, is limited. Further, physical and psychological conditions remain in silos based on an oversimplified approach to studying diseases that dichotomize the mind and body [12, 13, 17]. The 22 chapters of the ICD-10 are organized according to organ systems, and one chapter is devoted to mental and behavioral disorders [18]. Thus, the nosologies are destined to remain arbitrary within the limitations of the existing science and are based on operational rather than theory-based diagnostic criteria [6, 10, 12, 13].

Consequently, some authors have advocated for an epidemiological approach to identifying common patterns of cooccurrence that will offer directions for further rigorous investigation of etiology [19]. The idea is to employ an *a fortiori* approach using observed morbidities and estimate if these conditions cooccur beyond random chance or expected rates of overlap [11, 19]. Other authors warn that *a fortiori* categorization or use of observational variables to arrive at taxon disease entities is unlikely to be useful or may even be harmful [2] and advocate for a more direct investigation of etiological factors that can distinguish taxon disease entities from regions of artifact [2]. Specifically, Drake warns that too much reliance on manifest symptoms can lead to “confused and confusing attempts” to classify and treat diseases [20] resulting in diagnostic proliferation and unnecessary polypharmacy. 

### 2.3. Problem Number 3—Dynamic Patterns of Relationships

As noted earlier, the simultaneous presence of multiple health conditions is termed as comorbidity when there is an index condition as well as other distinct conditions and as multimorbidity when no single condition is identified as an index disease [4, 14]. An index disease refers to a condition or core mechanisms with relatively large impact on the development of comorbidity, its course and outcomes [21]. While operationally appropriate, the above conceptualizations undermine the dynamic and heterogeneous nature of comorbidities. 

**Figure 3:**
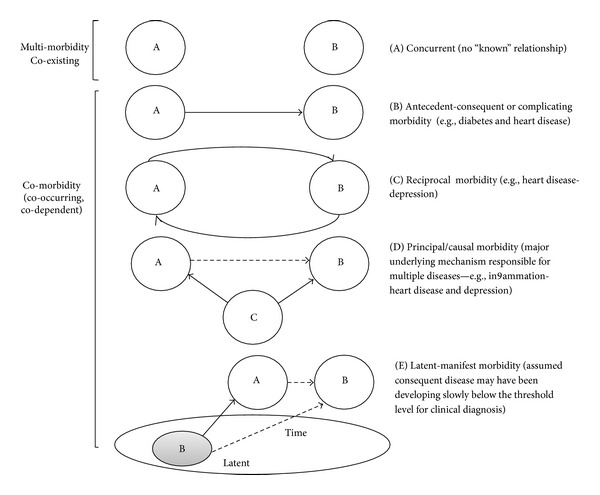
Dynamicity: patterns of relationships and complexities (illustration of conceptual problem no. 3).

C. van Weel and Schellevis [22] proposed four categories to capture the complex relationships among disease entities: (1) causal (diseases with a common pathophysiology), (2) complicating (disease-specific complicating morbidity), (3) concurrent (coexisting chronic morbidity without any known causal relation to the index disease), and (4) intercurrent (referring to interacting acute illness, usually limited in time). Based on evidence, additional levels of complexities can be introduced in the above categorization (see Figure 3). The *antecedent-consequent *(Figure 3(B)) category may be further confounded by the evidence of *reciprocal *(Figure 3(C)) and bidirectional association between diseases [23, 24]. For instance, heart disease and diabetes may increase risk for depression and depression may in turn increase the risk for cardiovascular disease and diabetes [19, 21]. Further, several antidepressants have anti-inflammatory properties and anti-inflammatory agents such that cyclooxygenase-2-selective inhibitors have been found to offer mood stabilizing benefits [21] suggesting presence of a *principal or causal* (Figure 3(D)) underlying mechanism responsible for multiple clinical conditions. Similarly, complicating morbidity, as proposed by van Weel and Schellevis, assumes an antecedent temporal relationship of an index disease to a consequent disease which may be arbitrary. This is because a *latent-manifest* (Figure 3(E)) relation among diseases is plausible; often clinical diagnoses are based on manifest indicators whereas an assumed consequent disease may have been present for years below the threshold level for clinical diagnosis [2]. Further, the relationship among diseases is complicated by other variables often neglected in comorbidity conceptualization and measurement such as stage, severity, complexity [6], health status, frailty, disability [25], and differences across socioeconomic, racial, gender, and age groups [8]. The specific interactions among risk factors and diseases may have synergistic, additive, or multiplicative effects on outcomes [14, 19].

Adding further levels of complexity in the understanding of patterns of relationships between diseases is the classification of diseases as distinct entities when in fact they may share underlying genetic homogeneity. Our human genome (the composite total of all of our chromosomes) is 99.9% identical within each individual; however, the genome is so large that there are about 3 million ways in which we can differ from one another [26]. Some of these differences predispose individuals to disease susceptibility. This is compounded by epigenetic factors, or our risk exposures in life, in which genetic expression can be altered [27, 28]. In effect, the impact of these genetic alterations can lead to health aberrations that manifest throughout multiple body systems, thus contributing to underlying comorbid pathophysiological processes. 

### 2.4. Problem Noumber 4—Atheoretical Understanding of Causes and Predictors

An understanding of the causes encompassing both the mechanisms and explanations of those mechanisms and predictors of unique patterns of comorbidities is the least developed area in the comorbidity literature. In an empirical review of causes and consequences of comorbidity, only 4 of 82 studies were concerned with the predictors of comorbidity [14]. This finding was supported by a more recent review of empiric comorbidity studies which found a similar lack of measurement of predictors of comorbidity [29]. Studies of social, environmental, and lifestyle risk factors are particularly lacking in the literature [14, 23, 30]. The paucity of research on antecedents versus consequences is understandable since consequences are relatively more straightforward to investigate and more rewarded in the prevailing culture of outcome-based medicine. In contrast, understanding etiologies represents a nearly insurmountable task of understanding a complex interplay of genetic, epigenetic, biological, neurochemical, structural, social, environmental, and situational risk factors with other intervening mediating and moderating variables [2, 4, 12, 16, 23]. Nevertheless, the predictor-based approach to understanding comorbidities is important since the risk factors for comorbidities are not randomly distributed in the population [4]. 

Further, there is a lack of critical use of certain variables as predictors of comorbidities. The variable of age is a case in point. Epidemiological and registry data for various incident diseases suggest that a number of comorbidities increase with age [4, 14, 31, 32]. It has been suggested that *aging* is related to increased burden of comorbidity and that comorbidity will only increase in the face of increasing life expectancy [4, 9]. Others have taken a more critical approach to understanding the effect of age and have argued that the disproportionate representation of certain age groups may result in conflicting conclusions on the relationships between age and comorbidity outcomes. For instance, Firat et al. [31] note that selection bias based on age in clinical trials precludes an understanding of the influence of age on comorbidity-related outcomes. Using an example from Dajczman et al. (as cited in Firat et al.), illustrated that patients 70 years or older are systematically excluded from cancer clinical trials. Only 1 of 81 patients older than 70 years of age was treated as part of an investigational protocol for small-cell lung cancer despite the evidence that a number of “fit elderly” can tolerate cancer treatment protocols. Nonetheless the protocol developed based on the age discrepancy is often accepted as the standard of care for all age groups [31]. In contrast, a Radiation Therapy Oncology Group (RTOG) study of concurrent chemotherapy and radiation therapy among “fit elderly” patients demonstrated greater survival benefits in older persons when compared to younger patients, illustrating that age may not be categorically associated with comorbidities and outcomes [31]. 

The quandary of age as an antecedent to comorbidity and outcomes remains. Most authors have used an aging society to underscore the significance of comorbidity work, and others have explicitly described age as an independent risk factor for comorbidities and multimorbidities [4, 6]. Stochastic risk factors that contribute to diseases increase with age (e.g., environmental exposure to toxins, passive smoking, and acquired genetic changes). Nevertheless, most studies have investigated the “on-average” effect of age and the risk may be different for subgroups of healthy-aged patients. 

## 3. Errors in Measurement

 Measurement influences everything currently known about comorbidity. The presence of comorbidity causes two related difficulties: one statistical and one clinical. The statistical problem arises from a failure to classify and analyze patients and their multiple diseases correctly resulting in misleading, therapy-related, mortality data in populations and individuals [1]. Clinically, comorbidity creates difficulties for both patients and clinicians. For the patient, anticipated outcomes from the index disease may differ based on the amount or type of comorbidities present. For the clinician, diagnosis becomes problematic as the index disease and comorbid disease may share signs and symptom patterns [33] complicating the evaluation of treatments [1]. 

The statistical difficulties, however, have driven comorbidity measurement from the beginning [1] and continue to exert an undue influence to today [3, 8, 34]. In clinical research the focus has been on controlling for comorbidity rather than understanding the particular condition and the implications for the patient. In the mid-1970s, Kaplan and Feinstein [33] grappled with taxonomic problems in classifying diabetes as a disease entity. In the 1980s, Charlson and colleagues [35] developed a comorbidity index to prospectively identify patients at greater risk of death during clinical trials. This focus on controlling for comorbidity continues on into the digital age with the development of measures utilizing ICD-10 codes [36–38] to aggregate large amounts of administrative data to analyze mortality and resource utilization patterns. For example, de Groot and colleagues [34], in a widely cited critical review of comorbidity measurement methods, listed four key reasons for careful measurement—confounding, effect modification, prediction, and efficiency. All four reasons are statistical rather than clinical and none are patient centered. But despite this focus on statistical precision there remain critical problems which result in errors in the measurement of comorbidity, primarily related to the presence of heterogeneous data sources, *atheoretical* summary measures, and outcome selection bias.

### 3.1. Problem Number 1—Heterogeneous Data Sources

The current identification of comorbidities is generally based on multiple data sources (administrative data sources, medical record review, clinician judgment, and patient interview) [29], each with advantages and limitations [8]. For instance, there are known limitations in using diagnostic data from ICD-10 codes, Current Procedural Terminology (CPT) codes, and discharge diagnoses as they pertain mainly to intensity and currency of services used which may exclude chronic diseases that are self-managed or pharmacologically managed. Other challenges in using ICD codes include “up-coding” (assigning a disease code associated with better payment) [36], underreporting (including only the number of secondary disease codes allowed by the database) [37], and failing to distinguish comorbidities from complications of care or severity indicators for the index disease [38]. Similarly, the use of pharmacy databases to ascertain comorbidities suffers from limitations, since participants may not have uniform access to drugs or may fill their prescriptions at pharmacies other than the one housing the database [8]. Using medical records as a data source requires the availability of longitudinal data to assure accurate construction of a comorbidity index. Even when available, the quality of data may vary by setting (inpatient versus outpatient) [8], format (electronic or paper-based), and quality of documentation across providers. Further, certain populations such as minorities, poor, uninsured/underinsured, elderly, and cognitively impaired are at higher risk for poor quality of diagnosis and documentation [4, 8]. Similarly, self-reported measures are limited due to risk of recall bias and may yield heterogeneous data due to variability in patient reporting. 

### 3.2. Problem Number 2—Atheoretical Summary Measures

Regardless of the data source, the majority of comorbidity measures are summary or aggregate measures. Although summary measures are important in quantifying the effect of an overall disease burden on outcomes, the greatest liability of summary measures is that they do little to advance the science underpinning comorbidities. Diseases occur on a continuum from presence of risk factors, subclinical disease to clinically detectable disease, progressing to multiple stages of advancement. Some elements of this disease continuum may not be amenable to direct observation or measurement. The “dynamic” component of disease progression, including the severity and rate of progress is beyond what most comorbidity instruments are able to capture. The operationalization of a principal or index disease is often based on investigator's lens and serves the purpose of contextualization. Thus the majority of the existing comorbidity measures are atheoretical and consequently limited in their ability to prognosticate [9] and advance science on diagnostic consolidation or common treatment approaches. 

Further, the existing measures and data sources may have varying levels of sensitivity for specific outcomes. For example in a study involving adults with lung cancer, comorbidity was related to survival outcomes when measured with the Cumulative Index Rating Scale. However, no association was found when the same outcome was measured using the Charlson Comorbidity Index [31]. It is also important to note that the most frequently used comorbidity measures are generic and often measure “multimorbidity” or “coexisting diseases” rather than comorbidities. Such measures may be appropriate for and sensitive to generic endpoints but not necessarily useful for disease- or treatment-specific outcomes.

### 3.3. Problem Number 3—Outcome Selection Bias

The outcome of comorbidities can be classified as generic (e.g., functional status, mortality) or disease-specific. Similar to the designation of an index disease, outcomes of comorbidities depend directly upon how they are identified, operationalized, and measured in a given study. Unlike generic outcomes that cut across diseases and comorbidities, disease-specific outcomes typically necessitate specific operationalization and tailored measurement. Disease- and treatment-specific outcomes have been noted to affect course and progression of illness [19], tolerance and response to treatment and treatment-related complications [19, 31], and behavioral outcomes [23]. It should be noted that outcomes in treatment-specific “efficacy” trials are known to be influenced by patient selection [31]. The presence of multimorbidity or certain types of morbidities is frequently exclusion criteria in efficacy trials [4, 11], which in turn may affect the study of treatment response, tolerance, and survival outcomes in the multimorbid. 

A further example of potential selection bias was illustrated in the comprehensive review conducted by Gijsen and colleagues [14] which concluded that while comorbidity typically affected health outcomes across study designs, settings, and outcomes with the effect remaining after adjustment of relevant confounders; that mortality, functional status, and quality of life were disproportionately reported as consequences of comorbidity. Interestingly, psychiatric comorbidities were found to be significantly associated with poor functional status or quality of life, whereas physiologic morbidities were associated with mortality. This may be artifact as effects of psychiatric comorbidities on outcomes such as mortality and health care utilization were seldom studied. In the few studies that investigated mortality outcomes in psychiatric comorbidities, mental disorders increased the risk for mortality both from suicide and having a comorbid disease [14]. The strong link of mental illness to worsening of physical health, illness burden, and premature mortality continues to be documented [19, 39]. 

From a health service perspective, comorbidities have been found to be consistently related to healthcare utilization and fiscal outcomes including cost, length of hospital stays, and number of physician visits [4, 14, 29]. In addition, there is evidence of interaction between age and patterns of utilization [4, 14] such that higher numbers of comorbidities in older adults are associated with more visits to both specialists and primary care providers (PCPs). In contrast, in the populations under 65 years, a greater number of specialist visits than PCPs visits were observed across all morbidity burden groups [4, 14]. 

## 4. Discussion

**Figure 4:**
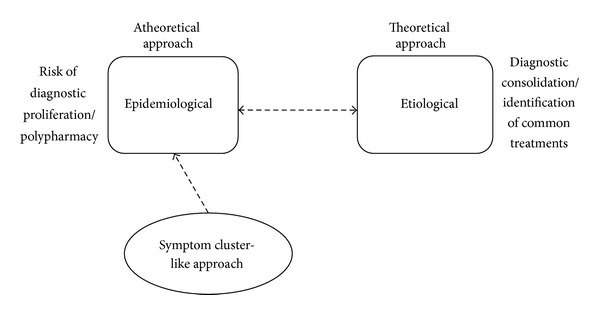
Closing the gap: translation of comorbidity science.

Despite accumulating literature, effective models for understanding, measuring, and addressing comorbidities are lacking. The two major approaches in the literature to address comorbidities are epidemiological and etiological (Figure 4). The epidemiological approach requires identifying disease entities that cooccur beyond random chance. This approach is purported to be “atheoretical” with accompanying risk of polypharmacy and diagnostic proliferation [4, 12, 13, 15, 40]. However, the etiological approach is in its rudimentary stages of development possibly due to intractability of identifying risk factors, shared mechanisms, interactions, and outcomes around any combination of diseases. New conceptual models are needed informed by an understanding of patterns of relationships among clinical and subclinical disease entities and mechanisms. 

Further, current barriers to moving towards a theoretical conceptualization of comorbidity need to be addressed. For instance, while many studies have been conducted to understand the consequences of comorbidities and multimorbidities, little research exists on common etiology and combined risk factors [14]. Specifically lacking are studies of psychological, environmental, and life-style risk factors [11, 14]. Some authors have cautioned that the debate around comorbidities should not be framed such that it leads to overvaluing of physiologic and pharmacological theories at the risk of undervaluing social etiologies [19, 20]. For instance, conceptualizing comorbid mental illness and substance use disorder as merely physiological may undermine the sociohistorical etiology of this relationship brought about by the closing and downsizing of mental health facilities [20]. 

Another barrier precluding research advancement relates to operationalizing comorbidities based on historical disease classification systems. Despite considerable evidence that psychiatric diseases are frequently accompanied by physical morbidities [41], researchers studying comorbid mental illnesses frequently fail to employ measures of physical morbidity and vice versa. 

Furthermore, failure to investigate comorbidities in a manner that captures patient's clinical complexity is another factor precluding advancement of comorbidity science. Investigators frequently exclude patients with complex comorbidities from clinical trials and fail to identify, report, and account for comorbidities in research even when they are present [4]. These research limitations may help to explain the clinical variability observed when protocols based on disease-specific clinical trials are applied to people with complex comorbidities [4]. Starfield [4] urged that rather than excluding patients with complex morbidities, participants in clinical trials should be characterized according to their total morbidity burden including patterns and types of illnesses followed by subgroup analysis to understand variability in outcomes based on morbidity burden. 

### 4.1. Recommendations for Addressing Conceptual and Measurement Errors

Rigorous studies that capture the complexity of comorbidities and its etiology from social, psychological, and biological perspectives are needed (Table 1). Researchers should carefully consider potential design and measurement issues in designing studies. For instance, the ascertainment of comorbidities depends upon both correctly applying diagnostic algorithms and accurately recording diagnostic data. This, in turn, depends upon factors including clinician's judgment, diagnostic skills, as well as accurate and complete documentation in the medical records. Special attention should be paid to inclusion of vulnerable populations such as minorities, poor, uninsured/underinsured, elderly, and cognitively impaired people who are disproportionately affected by ascertainment bias [4, 8].

While correct ascertainment of comorbidities and minimizing sources of errors are important, these corrections do not address the problems related to measurement. An area in need of critical debate is the scientific approach to addressing measurement gaps. The classical psychometric approaches seek to identify consistency and homogeneity in a phenomenon while “dehumanizing” clinical data to make it amenable for statistical analysis [11, 42]. Psychometric models alone may not be appropriate for a heterogeneous and dynamic concept like comorbidities. 

## 5. Limitations

Certain limitations should be acknowledged in this paper. This review was not meant to be systematic and exhaustive; rather the purpose of this paper was to understand the current fluid discourse and directions on comorbidities, which research literature often misses due to focusing on a narrow set of (often disease-specific) variables under study. Thus nonempirical literature (reviews, editorials, and commentaries) was deliberately selected to allow a more inclusive understanding of this topic. Also, we limited our literature search terms to the words “comorbid,” “comorbidity,” and “comorbidities” in the title, which defined the pool of included papers. Use of different search terms in different databases may have resulted in the identification of additional papers not included here. This should be kept in mind when examining the findings. However, based on our experience with an earlier systematic review conducted by members of this group [29], we are confident that this search captured the salient papers related to current interprofessional discourse surrounding comorbidity.

## 6. Conclusions

Extant literature suggests that the nature of comorbidities is defined by an evolving nosology, dynamic and heterogeneous interactions between and among disease entities that may not follow a clear antecedent-consequent relationship nor linear-temporal progression. The index designation may be arbitrary in the absence of strong etiological science. The consequences of comorbidity may be categorized as generic (e.g., functional or fiscal) or disease-specific. Of particular concern, the professional discourse is remarkably unchanged from the early conceptual work [1] to today [11].

The goal of comorbidity science is to identify accurate patterns of relationships between and among disease entities and to reliably characterize their relationships to risk factors and outcomes. Many questions remain in the conceptual and measurement domains: What are the essential dimensions of comorbidities? Is the current conceptualization in the context of a distinct additional clinical entity serving as a red herring distracting from the advancement of the conceptualization of comorbidity? Should subclinical diseases, presence of mechanisms (e.g., inflammation or oxidative stress), and health states (e.g., frailty, and cognitive and functional status impairments) be considered dimensions of comorbidities? Does a comprehensive index of clinical states and symptoms serve as a better indicator of outcomes than disease states? What are the valid approaches to advancing knowledge of comorbidities; and what should be the starting point or anchor for these developments (e.g., types of diseases, types of outcomes, or type of populations to be studied, such as age groups)? 

Thus, the concept of comorbidities and its measurement is in need of meaningful debate by the scientific and clinical communities. In the meantime, comorbidities should be contextualized as an emerging science rather than biological realities or “scientific givens” [20]. It is important to be aware of the real risks of overtreatment and overemphasis of medical interventions while underutilization of social and behavioral interventions towards prevention and disease management [20]. Researchers, clinicians, and policy makers are urged to draw careful conclusions and implications in interpreting comorbidity research findings. 

## Figures and Tables

**Table 1:** Recommendations for improving comorbidity conceptualization and measurement.

Domain	Directions
Conceptual	(i) Carve valid next steps with integrated input from clinicians, researchers, taxometricians, psychometricians, and patients. (ii) Develop complex conceptual models that capture the complexity of comorbidities while moving away from mind-body, organ system dichotomies. (iii) Conceptualize comorbidities in a manner that encourages investigation of both biological and social etiologies of comorbidities and outcomes. (iv) Keep the patient at the center of all conceptualization endeavors. (v) Build the science from both epidemiological and etiological perspectives in tandem.

Research	(i) Design rigorous longitudinal comorbidity mapping projects that also collect comprehensive data on sociodemographics, lifestyle factors, environmental factors, biomarkers, and outcomes. (ii) Commit resources and funding (directed RFAs and supplements from major research agencies and across institutes). (iii) Bridge gaps in understanding of physical and psychiatric morbidities. (iv) Leverage epidemiological and statistical approaches to move comorbidity science from atheoretical to theoretical; that is, model various sources of uncertainty (e.g., multiple bias modeling), amplify data through simulation (Monte Carlo techniques), and improve understanding of relationships among variables (e.g., using Hybrid Structural Equation modeling techniques combining observed and latent variable analysis). (v) Interpret carefully findings within the limitations of an emerging science rather than as biological realities or “scientific givens”. (vi) Educate clinicians, researchers, and policymakers about the risks of atheoretical approaches to understanding comorbidities including diagnostic proliferation, polypharmacy, and cost. (vii) Include populations such as minorities, poor, uninsured/underinsured, elderly, cognitively impaired, and those with complex morbidities who are disproportionately excluded from comorbidity research.

Measurement	(i) Characterize and minimize potential sources of erroneous inference. (ii) Develop measures that capture complex and dynamic nature of comorbidities beyond numbers and severity of diseases. (iii) Incorporate a discussion on nontraditional measurement approaches such as *clinimetrics* in conceptualizing measurement (e.g., approaching comorbidities using a battery of psychometric and clinimetric instruments that address different dimensions of the phenomenon such as types, severity, trajectory of diseases or symptoms, and rates of progression, clinical states such as functional capacity, and other aspects of health such as well-being and distress). (iv) Develop measures that combine physical and psychological morbidities.
